# How Can Genomic Innovations in Pediatric Brain Tumors Transform Outcomes in Low- and Middle-Income Countries?

**DOI:** 10.1200/GO.22.00156

**Published:** 2022-10-17

**Authors:** Simon Bailey, Alan Davidson, Jeannette Parkes, Uri Tabori, Anthony Figaji, Shridar Epari, Girish Chinnaswamy, Rosdali Diaz-Coronado, Sandro Casavilca-Zambrano, Nisreen Amayiri, Gilles Vassal, Eric Bouffet, Steven C. Clifford

**Affiliations:** 1 Great North Children's Hospital and Newcastle University, Newcastle upon Tyne, United Kingdom; 2 Haematology Oncology Service, Red Cross War Memorial Children's Hospital, Department of Paediatrics and Child Health, University of Cape Town, Cape Town, South Africa; 3 Department of Radiation Oncology, Groote Schuur Hospital and University of Cape Town, Cape Town, South Africa; 4 Neuro-oncology Program, Division of Haematology/Oncology, The Hospital for Sick Children, Toronto, ON, Canada; 5 Department of Neurosurgery, Red Cross War Memorial Children's Hospital and University of Cape Town, Cape Town, South Africa; 6 Department of Pathology, ACTREC and Tata Memorial Hospital, Tata Memorial Centre, Homi Bhabha National Institute, Mumbai, India; 7 Department of Pediatric Oncology, Tata Memorial Hospital, Parel, Mumbai, India; 8 Pediatric Oncology Department—Instituto Nacional de Enfermedades Neoplásicas, Surquillo, Peru; 9 Instituto Nacional de Enfermedades Neoplásicas, Lima, Perú and Facultad de Ciencias de la Salud de La Universidad de Huánuco, Huánuco, Peru; 10 Department of Hematology and Oncology, King Hussein Cancer Centre, Amman, Jordan; 11 Department of Pediatric and Adolescent Oncology, Institut Gustave-Roussy, Villejuif, France; 12 Wolfson Childhood Cancer Research Centre, Newcastle University Centre for Cancer, Newcastle upon Tyne, United Kingdom

## INTRODUCTION

Advances in molecular diagnostics have led to improved stratification and targeted interventions in the treatment of children with brain tumors. This has necessitated complex infrastructure to deliver all the required testing in a clinically useful time period. However, in less-resourced countries, this testing is not routinely available and an ever-widening gap in the ability to deliver more tailored therapies including targeted agents is increasingly evident. This article reviews the recent advances and suggests practical ways of ensuring that genomic advances are applied according to available resources.

**CONTEXT**

**Key Objective**
To suggest how genomic innovations can be used to improve outcomes for children with CNS tumors in low and middle income countries (LMICs) and to put these innovations in overall context in terms of care of these children.
**Knowledge Generated**
Eighty percent of the CNS tumors found in children are in LMICs, but they have reduced access to both genomic testing and any targeted therapies used as a result of these tests. Clinical teams in LMICs, however, endeavor to provide the best for their children with CNS tumors despite the limitations.
**Relevance**
Selective testing and the introduction of clinical trials as well as support of institutions from high-income countries and pressure on pharmaceutical companies and richer governments to provide newer targeted agents and technological support will enable children with CNS tumors in LMICs to have improved outcomes.

## CLASSIFICATION

The WHO classification of brain tumors (2021)^1^ now includes molecular findings in a multilayered approach to diagnosis. Although a certain level of diagnostic information is essential for basic entity recognition and treatment planning in most diseases, other information (eg, information required to support the delivery of risk-stratified adjuvant therapies and advanced or biomarker-stratified targeted therapies) may be considered nonessential in clinical settings where such therapies are not routinely delivered. As a result, the WHO classification allows not otherwise specified diagnosis for most tumor types.^1^

Testing for common molecular disease groups, mutations, amplifications, or fusions that lead to risk-adapted or targeted therapies requires additional testing methodologies, most of which are not routinely available in low- and middle-income countries (LMICs). The most common of these are presented in Table 1. Detailed testing currently may or may not, depending on the region, lead to change in therapy as drugs are often not available.

**TABLE 1:**
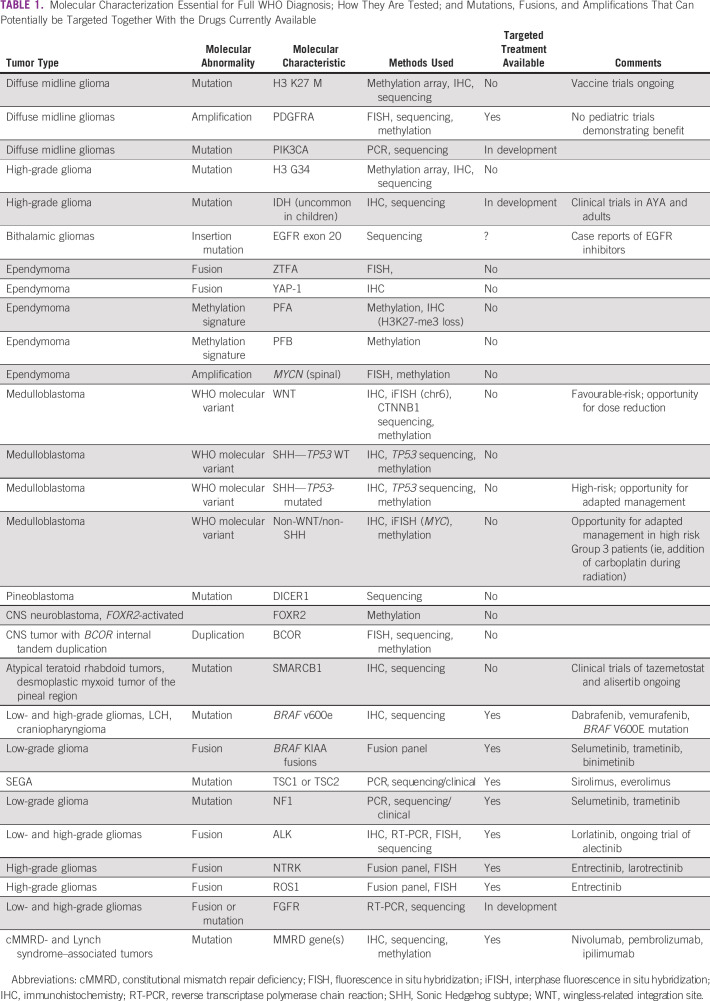
Molecular Characterization Essential for Full WHO Diagnosis; How They Are Tested; and Mutations, Fusions, and Amplifications That Can Potentially be Targeted Together With the Drugs Currently Available

## BRAIN TUMOUR DIAGNOSIS AND MANAGEMENT IN LMICs

Each year, approximately 429,000 children (age 0-19 years) are affected with cancer, of which approximately 90% are from LMICs.^2^ The cure rate in high-income countries (HICs) exceeds 80% but is < 30% in LMICs. Similarly, the majority of children presenting with CNS tumors live in LMICs, but data on the incidence, survival, and burden of CNS tumors are poor, even when compared with other childhood cancers.^3^ According to the CONCORD working group, 5-year survival from brain tumors in children is higher than that for adults, but the global range is very wide (28.9% in Brazil to nearly 80% in Sweden and Denmark). However, this survival range does not depict the actual situation in low-income countries where national registries do not exist and publications are few.

The reasons for the survival gap between HICs and LMICs are many and complex including underdiagnosis, delayed presentation, and unavailability or inaccessibility of multidisciplinary neuro-oncology treatment facilities including neurosurgical and radiotherapy equipment. Seah et al^4^ reported on abandonment of treatment for pediatric CNS tumors and concluded that failure to start or complete potentially curative therapy is also a key contributor to poor outcomes.^5^

Very little data are available from most low-income countries. In Sudan, Elhassan et al^6^ report 2-year and 5-year survival rates of 33% and 13%, respectively, in a series of 62 patients with pediatric brain tumor and also attribute this to underdiagnosis, inadequate treatment, and treatment abandonment. A gradual increase in numbers of cases diagnosed is noted since 2000, but only 60% of CNS tumors are diagnosed on the basis of biopsy.

St Jude Children's Research Hospital recently launched a Global Academy Neuro-Oncology Training Seminar, focused on LMIC needs in pediatric neuro-oncology. The group identified the following as barriers to care: (1) an absence of coordinated multidisciplinary care; (2) an inability to subspecialize or concentrate on neuro-oncologic diseases; (3) limited infrastructure, including neurosurgical, laboratory, radiotherapy, and rehabilitation facilities; (4) delays in referrals between specialties; (5) postsurgical morbidity; (6) insufficient hospital-based and population-based data; (7) treatment abandonment; and (8) an increasing discord between recent molecular insights and the current clinical context in LMICs.^3^

## MOLECULAR TESTING AND REQUIRED INFRASTRUCTURE

The implementation of molecular testing, and the prioritization of testing infrastructure, should be pragmatically driven by multiple inter-related factors, including test availability, diagnostic infrastructure capability, technical expertise, and an understanding of the relevance of specific tests to affect clinical management decisions. Together, these must be tailored to the level of resources available to support these activities.

Standard histologic and immunohistochemistry-based diagnostics, which encompass morphological and phenotypic features assessable using conventional histology techniques, and immunostains, which are deliverable in most institutions; typically, these will enable diagnosis of histologic variants using WHO criteria, with support from histopathology second opinion referral networks where required.

Second, enhanced histologic diagnosis, where further specific immunostains are used as associated or surrogate features of specific key molecular events to aid differential diagnosis. Examples include recognition of *BRAF* mutation–associated staining in pilocytic astrocytomas and the wingless-related integration site (WNT), Sonic Hedgehog subtype (SHH), and non-WNT/non-SHH medulloblastoma disease groups. Although these are indicative and useful adjuncts to histologic diagnostics, they would not typically meet standards for the definition of molecularly defined WHO variants.

Third, combined histologic and genetic testing, where specific critical genetic lesions may be assessed using low-throughput technologies such as interphase fluorescence in situ hybridization for chromosomal defects or Sanger sequencing for specific mutations. For many diseases, such approaches will allow the definition of WHO-defined entities at the molecular level and may be delivered through the implementation of basic genetic laboratory services. In 2022, this likely represents the majority of practice in high-income countries.

Finally, fully integrated molecular diagnostics and pathology review, which assess all biologic disease features and provide expert pathology review, to support all differential diagnoses. Typically, this will include next-generation omics technologies (eg, panel or whole-exome/genome sequencing and DNA methylation array), which detect lesions across the genome in addition to those specifically required, and cross-validation of specific molecular findings across different alternative techniques. These are typically delivered in highly specialized centers and, although they have greatest cost implications, are also most cost-efficient in terms of information delivered per unit cost. Today, such systems are typically in place in national specialist networks of centers within high-income countries and may support diagnostics requirements for biomarker-driven clinical trials.

Wider considerations in the selection of molecular testing and pathology review approaches will include potential for centralization, whereby multiple local treatment centers refer to a specialist center for assessment on behalf of local, regional, national, or international networks. Such networks may allow efficiencies of cost, throughput, and centralization of key expertise and include central pathology review.

Sampling methodologies and turnaround times are also major inter-related factors. The level of testing, which can be delivered, will depend on the amount and quality of tumor material available. Training may need to be provided for preparation (fixation, sectioning, and staining) of good-quality histologic slides. Although histology-based testing may be routinely accomplished on formalin-fixed paraffin-embedded material, advanced genomics methods typically require snap-frozen material stored at −80°C. Moreover, any diagnostics infrastructure must be able to deliver integrated results to an multi-disciplinary team (MDT) including treating clinicians in a timely fashion to enable therapy selection and planning, before commencement of adjuvant therapies (typically within 3-4 weeks of presentation). In particular, any centralized system adopted must be compatible with these timescales and able to collect good-quality tumor material.^7^

## CURRENT LMICs PRACTICE

**FIG 1:**
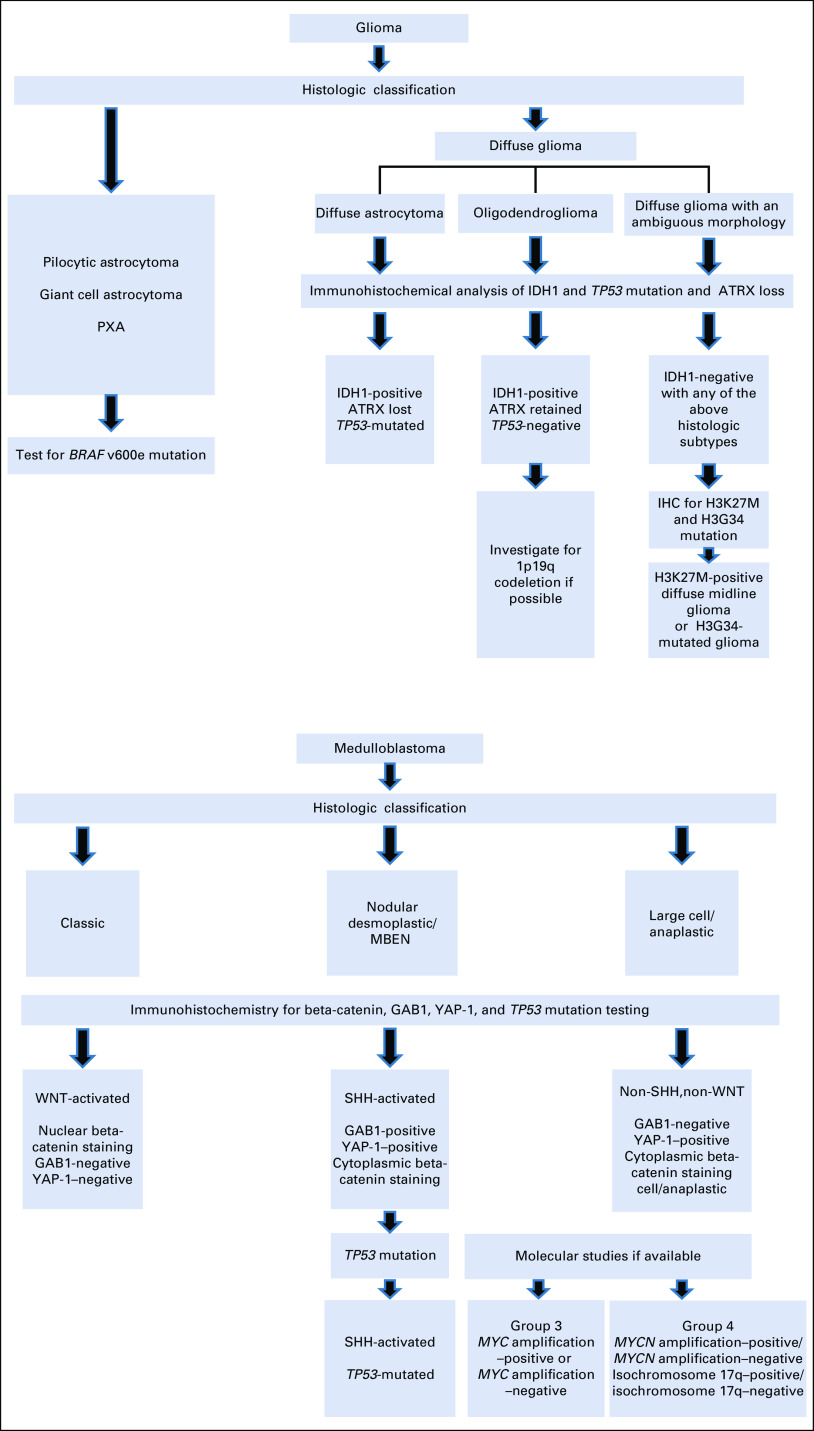
Examples of resource-stratified diagnostic algorithms using immunohistochemistry for LMICs. IHC, immunohistochemistry; LMICs, low- and middle-income countries; MBEN, medulloblastoma with extensive nodularity; PXA, pleomorphic xanthoastrocytoma; SHH, Sonic Hedgehog subtype; WNT, wingless-related integration site.

The WHO classification of brain tumors (2021)^1^ proposes a new vision in the diagnosis of CNS tumors, integrating molecular classification as an element to be considered in the histologic grade, as proposed by the publications of the cIMPACT-NOW working group. It is in this context that a complex reality arises and strategies are required so that developing countries, which, in general, have technological and human resource limitations for molecular diagnosis, can have access to technologies that allow the optimization of resources to implement an integrated diagnosis that allows individualized management and access to therapeutic advances. Such tailored approaches have been developed in a number of LMICs/low-income countries (LICs), and we present one such approach (Fig 1) although the majority of LMIC centers do not yet have access to these techniques. The major developments have been in the field of gliomas and embryonal tumors, and with the use of immunohistochemistry and carefully selected molecular tests, clinically useful information that affects treatment decision making can be ascertained.

The detection of molecular targets (Table 1) does not always equate to clinical effectiveness, and it is important that potentially expensive therapies are shown to be effective before committing hard earned resources in accessing these agents. The majority of these agents are used as second-line therapy even in HICs; however, agents such as MEK inhibitors, which are clinically very useful in treating children with unresectable low-grade gliomas (LGGs), are often difficult to access in LMICs/LICs. Work is ongoing to add such agents to the WHO essential medicines list.^8^

## DRUG ACCESS IN LMICs

In addition to access to appropriate imaging, appropriate neurosurgery, and pediatric radiotherapy, easy access to anticancer drugs has been a major hurdle for delivery of cancer care in many parts of the world especially in LMICs. This applies to both adult and pediatric cancers. The disparity is not just limited to newer agents such as targeted/biologic therapies but even to conventional chemotherapeutic drugs. Even in areas where chemotherapeutic agents are available, the supply chain has been inconsistent, making continuity of care a real challenge.

The major reason for lack of access to these essential medicines is the unwillingness of drug companies to register older, cheaper drugs and the high pricing of new and off-patent drugs. This has led to the nonaffordability of these drugs in the majority of the population in LMICs. Coupled with this is the lack of wider universal health insurance coverage of the population and inadequate government funding to support such therapies.^9^

A technical report by the WHO revealed that there was a much lower availability of anticancer drugs in countries with a lower national income. The cancer drugs on the essential medicine list of WHO are available to 32% and 57% of LICs and middle-income countries (MICs), respectively, and importantly, only to individuals who can afford the cost of the drugs (as an out-of-pocket expenditure).^10^ A recent survey performed among medical oncologists globally revealed that around one third of conventional chemotherapeutic drugs were only available in LMICs by out-of-pocket expenditure. Such expenditure was commonly financially catastrophic for families when newer/targeted molecules such as rituximab and trastuzumab were required^11^ (the same group has conducted a survey for pediatric cancer within SIOP, and the results should be available soon).

Many of the targeted agents, which have been proven to be effective in early-phase pediatric brain tumor trials (eg, vemurafenib, dabrafenib, bevacizumab, and trametinib), have been licensed for use in adults in many countries.^12,13^ Even in some MICs where these drugs are available, the high cost continues to be a major hurdle for widespread use of these therapies. Moreover, these drugs need to be given for a long period of time, and the majority of studies have shown the duration of therapy to be over 1 year, which leads to a long-term problem with accessibility and affordability. Limited compassionate access programs by the pharmaceutical industry and paucity of clinical trials of such drugs in LMIC settings have made it even more difficult for children with brain tumors to access these targeted agents.^14^

Thus, the application of exciting developments in molecular diagnostics and identification of prognostic and predictive biomarkers in LMICs/LICs are not straightforward and each country/region needs to ascertain the clinical usefulness of introducing the infrastructure and support that is required within their financial constraints.

### Possible Solutions

#### Repurposing and use of low-cost diagnostic tools building up in the planned way to more technologically advanced solutions.

The current WHO classification of brain tumors^1^ adopts methylome-based diagnostics as the desirable avenue for precise classification and diagnosis. However, the methylome-based approach excludes many parts of the world because of lack of availability of the platform and inherent financial constraints. Thus, it is important to resort to an alternative integrated approach using clinicoradiologic and histologic features including immunohistochemistry for choosing an appropriate target gene/variant testing and arriving at a clinical useful diagnosis, some of which is discussed below. Barring a few entities like high-grade astrocytoma with piloid-like features, etc, which are essentially methylome-defined, most of the other entities are defined by certain specific molecular alterations, which can be tested using a histologic approach with supplementary specific molecular testing.

##### Embryonal tumors.

The commonest tumor of this group is medulloblastoma, which in the current WHO classification, contain molecularly defined subgroups (WNT-activated, SHH-activated and non-WNT/non-SHH) and histologically defined subgroups (classic, nodular desmoplastic, with extensive nodularity and large cell anaplastic, not otherwise specified—with myogenic differentiation and with melanotic differentiation). Molecularly defined groups are used in routine clinical practice and have been shown to have better predictive value than histologic subtypes alone.^15^ Although the gold standard method in defining subgroups is methylation profiling, there are more readily accessible molecular methods such as targeted gene expression (both real-time–based and NanoString^16^ or MIMIC technology^17^). If these tests are not available, algorithms using an immunohistochemistry panel of four to five antibodies with incorporation of fluorescence in situ hybridization–based evaluation for single gene copy number and clinicoradiologic findings can serve to provide an alternative to the more expensive molecular methods for molecularly defined subgroups of medulloblastomas^16-18^ (Table 2). Other embryonal cell tumors such embryonal tumor with multilayered rosettes (ETMR) and atypical teratoid rhabdoid tumors (ATRTs) can be diagnosed using appropriate immunohistochemistry markers of LIN28A and INI1, respectively. Most ETMRs show diffuse strong positivity for LIN28A in the undifferentiated component of ETMR architecture and diffuse strong positivity across the entire tumor in the ependymoblastoma-like architecture (can be supported by C19MC amplification testing if available),^18,22^ whereas the ATRTs are characterized by loss of expression for INI1 protein and in approximately 5% of cases, show retained INI1 protein expression with loss of BRG protein expression (can be supported my SMARCB1 genetic testing if available).^23^ Other embryonal cell tumors such as CNS neuroblastoma, FOXR2-activated, require diagnosis by the methylation array or more complex molecular methods.^24^

**TABLE 2:**
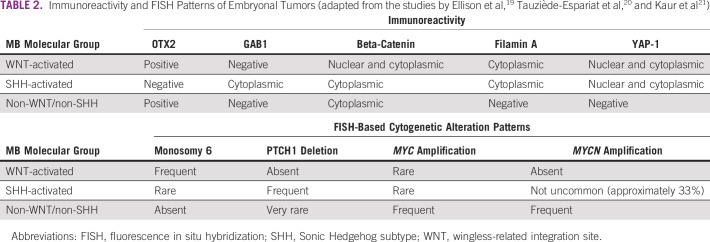
Immunoreactivity and FISH Patterns of Embryonal Tumors (adapted from the studies by Ellison et al,^19^ Tauziède-Espariat et al,^20^ and Kaur et al^21^)

##### Ependymoma.

Currently, these tumors are broadly classified as per the location—supratentorial (ZFTA fusion–positive and YAP-1 fusion–positive), posterior fossa (type A and B), and spinal (ependymoma, with *MYCN* amplification, myxopapillary ependymoma, and subependymoma) groups. Using immunohistochemistry for L1CAM in supratentorial^25^ and H3K27me3 protein in posterior fossa,^26^ further molecular grouping can be achieved in ependymomas. In the case of the spinal ependymomas with high-grade morphology, these can be screened with immunohistochemistry for *MYCN* to help in identifying the subset of spinal ependymoma with *MYCN* amplification^27^ (Table 3).

**TABLE 3:**
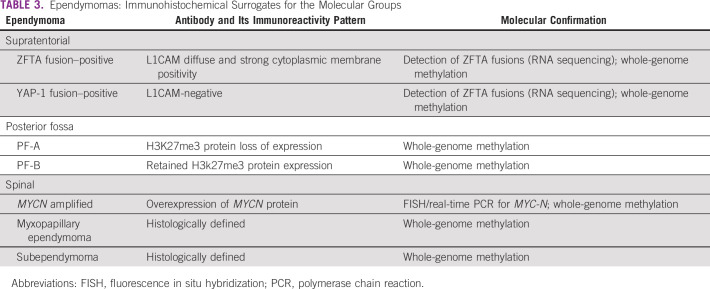
Ependymomas: Immunohistochemical Surrogates for the Molecular Groups

##### Glial tumors.

Most of the clinically relevant distinction of low-grade and high-grade gliomas can be done on histology and wherever indicated, with integration of radiologic findings. The majority of low-grade glial and glioneuronal tumors are fusion-defined although some are characterized by single-nucleotide variation and rarely by copy number variation. Strictly speaking, there are no alternative or immunohistochemical surrogates for these genetic alterations. Clinically, however, the entire gamut of pediatric low-grade glial and glioneuronal tumors are managed in the clinics with a similar protocol and the majority show typical histologic features, which can serve as a robust biomarker for these tumors and predict the involvement of mitogen-activated protein kinase pathway activation. There are now histochemical markers for many of these variants as outlined in Table 4.

**TABLE 4:**
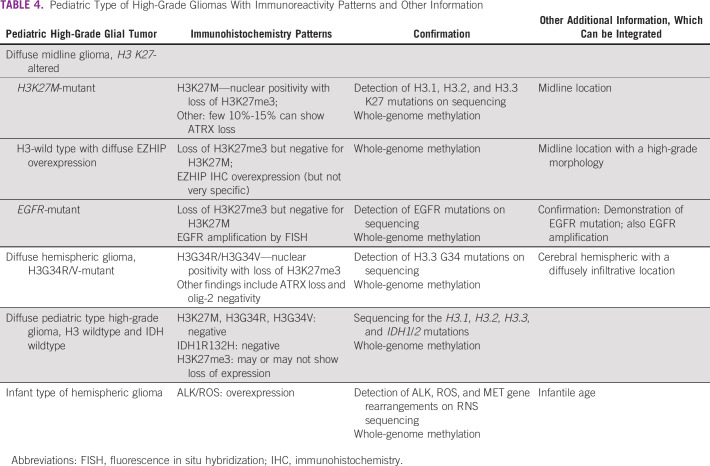
Pediatric Type of High-Grade Gliomas With Immunoreactivity Patterns and Other Information

#### Clinical trials in LMICs/LICs.

There has been a steady increase in reporting of the epidemiology and outcomes of pediatric brain tumors in LMICs over the past three decades. A survey of publications in comprehensive and recent review articles^28-31^ reveals a dearth of literature on pediatric brain tumors in LMICs before the turn of the century, with a significant and sustained increase after the year 2000. The publications emanate from Asia, Africa, Latin America, and the Mediterranean region, but most are retrospective audits and very few describe clinical trials in the sense of prospective evaluation of a predetermined treatment strategy. Only four published works appear to satisfy this requirement, and only one of them was published after the year 2010.^32-35^

There are no publications describing clinical trials in the LMIC setting of biologic agents commonly in use in HICs for pediatric brain tumors (vascular endothelial growth factor inhibitors, V600E-mutated *BRAF* inhibitors, MEK inhibitors, smoothing inhibitors, and cell cycle checkpoint inhibitors). There are, however, some excellent reports describing the utility of molecular subtyping in predicting the outcomes of medulloblastoma^19,36,37^ and at least one report demonstrating the feasibility of high-dose chemotherapy for infants with brain tumors.^25,38^ There are case reports describing the use of targeted therapies for individual patients on a compassionate basis in LMICs,^39,40^ and there are, encouragingly, two phase II trials ongoing in India: bevacizumab in DIPG (on the basis of perfusion patterns on magnetic resonance imaging [MRI]) and COMBAT therapy (using combination of valproate for histone deacetylase inhibition, retinoic acid, and temozolomide) for high-risk medulloblastoma (G. Chinniswamy, personal communication, 2022).

Development and implementation of trials should be encouraged and advocated, pressure should be put on pharmaceutical companies to expand their trials to less advantaged nations, and this may also require additional resource to build the necessary trial infrastructure. Clinical trials of newer agents would benefit the children and families in these regions and enhance the level of care that these children are able to access.

Pharmaceutical companies should be encouraged to provide financial and logistic support for relevant research.

#### Twinning and collaboration.

There is no doubt that twinning between HIC and LMIC centers and even between MIC and LIC centers can provide powerful intellectual and logistic support (such as remote/digital pathology, diagnostics, and radiology review) to the MDT in LMICs. The crucial first step is the creation of an MDT in the LMIC context. This could be as simple as a physician and an oncology nurse, but would ideally extend to involve the surgeon, the radiation oncologist, the pathologist and radiologist, and the whole ancillary team. Where MDTs in the LMIC setting are grappling with the setting of priorities and the interpretation of diagnostic information, the development of these relationships can provide crucial assistance as LMIC teams develop the capacity to manage those processes.^41,42^

#### Compassionate use programs.

Although some targeted therapies have many advantages that would benefit patients in LMICs, access to these medications is a major issue and costs are prohibitive for patients and families. Many of these agents are administered orally, and this would potentially avoid the need for peripheral or central venous access and minimize the costs and challenges associated with travel. Most targeted medications used in pediatric brain tumors are nonimmunosuppressive agents and do not cause a risk of neutropenic fever that would require a stay in the vicinity of the health care facility. However, reports on the use of targeted therapies for pediatric brain tumors in LMICs are rare and limited to case reports. A literature search could only identify two case reports from Jordan and Pakistan, both in relation to *BRAF* V600E–mutant gliomas.^39,40^ Although compassionate access took only one week in the first case, it took more than one year of persistent efforts in the report from Mustansir. In both cases, patients showed a remarkable response to targeted therapy with mild and manageable side effects.

#### Closing the gap in drug access.

The WHO essential medicine list for children (EMLc) does not secure access to medicines, but provides clear and validated guidance for all countries, governments, and health insurances on which medicines should be available for all children with cancer around the globe at all times. This is an important official tool to advocate and lobby in any country for specific actions at the government and policy-making level. Continuous efforts from the pediatric oncology community should be made to enrich and update the WHO EMLc. For instance, in 2021 WHO global initiative for Childhood Cancer,^43^ in which pediatric low-grade glioma—the most common brain tumor in children—is one of the six index diseases, it is critical to develop a large global program that will give children and adolescents with pediatric LGGs in LMIC access to targeted therapies. This will also require access to diagnostic techniques, as most methods used for the identification of molecular alteration are not available or too expensive in LMICs. Efforts are ongoing to develop more affordable techniques to detect molecular alterations and to partner with the pharmaceutical industry to initiate pilot studies that aim at demonstrating the feasibility and safety of targeted therapies in this setting.

### Examples of How Biology can Affect Treatment

Examples of tumor types where a more detailed knowledge of biological information enhances the treatment of those children can be found in Appendix 1 and Table 5. The tumor types discussed are LGG, medulloblastoma, and genetic factors/familial disease.

**TABLE 5:**
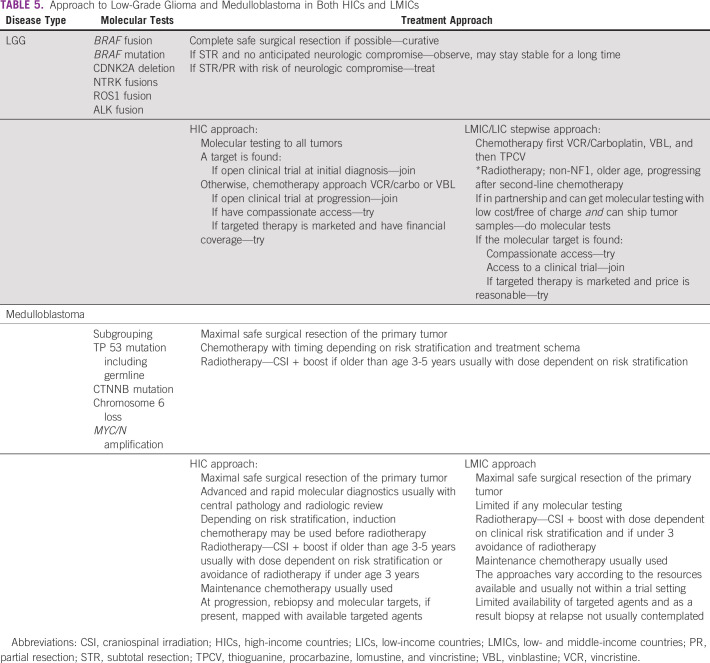
Approach to Low-Grade Glioma and Medulloblastoma in Both HICs and LMICs

## DISCUSSION

With advances in molecular diagnostics and the ever-increasing number of available targeted therapies, there is an ever-increasing gap between HICs and LMICs because of the differing ability to offer risk-stratified and targeted therapy. Where tumors cannot be identified as requiring less toxic or targeted treatment, there is potential for significantly increased toxicity for children who survive with a consequent reduction in lifelong quality of life, especially since supportive measures may be lacking in LMICs. There are no simple or rapid solutions to address this imbalance, but this review has attempted to offer some potential ways forward.

Molecular and biologic analyses are not all-or-nothing phenomena, and implementing testing using lower-cost alternatives can give clinicians important information in risk stratification. Examples of this approach have been discussed above.^19-21,25-27^ Implementing prospective molecular studies in a centralized national or regional manner would help to obtain timely results, and even retrospective analysis of these cohorts could help inform future trials in LMICs. These strategies must be aligned and carried out in a coordinated manner such as part of the WHO Global Initiative for childhood cancer, which has the aim of improving the survival of pediatric patients in LMICs to 60% by 2030 through early diagnosis, improvement of abandonment rates, increasing supportive treatments, and creating local capacity and policies. The application of more advanced diagnostics must be part of a multidisciplinary consensus within each country and region. Regional or national collaborative groups are key to generate clinically useful cost-effective and sustainable initiatives in an attempt to reduce the gap between HICs and LMICs.

Clinical trials (or at least standardized and audited treatment approaches) have many advantages to patients and their families; they generally result in better outcomes and allow standardized treatments with a view of improving outcomes.^44^ There are relatively few published clinical trials in LMICs in comparison with HICs. This applies to both academic and commercial trials and is usually due to limited resources, with clinical trials being expensive to run. Commercial enterprises are reluctant to run clinic trials in LMICs as they see a limited potential gain in terms of future revenue. Both commercial and well-funded academic institutions in HICs should be encouraged to sponsor trials led by the local and regional teams in LMICs.

Twining between centers in LMICs and HICs is useful in many ways but should be viewed as a two-way process benefitting both the LMIC and HIC partners.^45^ These should be led by the agenda and needs of the LMIC unit. Partner units may, under certain circumstances, be able to assist with identification of prognostic and predictive biomarkers and diagnostic testing unable to be performed in the LMIC. HIC units can help by encouraging and facilitating development in LMICs, resulting in long-term sustainability, and this includes the development of clinical expertise and experience especially with newer technologies and therapeutic agents. In addition, use of technology such as remote digital pathology, review of MRI scans, and teleconferencing for a HIC-/LMIC-combined MDT may be used. Organizations such as SIOP (International Society of Paediatric Oncology), ITCC (Innovative Therapies for Children with Cancer), Children's Oncology Group (COG), and other consortia can play a major role in applying political pressure on both organizations such as the WHO and Pharmaceutical companies to improve access to novel therapeutic agents in LMICs.

Compassionate use programs for some newer agents are available before regulatory approval in many HICs. Despite the potential to provide safe alternatives in disease such as LGG (one of the WHO six index pediatric tumors), compassionate use programs are generally not available. Companies should be encouraged and perhaps more controversially legislated to provide such programs before regulatory approval to disadvantaged nations. Political pressure should be applied to help those children and families in LMICs.

The WHO has performed a lot of good work in providing an essential medicines list to advocate at the country level for specific actions. However, the list is yet incomplete with regard to childhood cancers. Although treatment for LGG is now on the list, this has taken much concerted work from many people in the pediatric oncology community. Continuous efforts need to be made to enrich and update the WHO EMLc to include a wider range of pediatric CNS tumors and more targeted therapies.

LMICs have a range of resources, and there is not one solution that will work in all countries. Importantly, the application of genomic advances will differ from country to country and even from region to region or hospital to hospital. Advances in both patient care and treatment and more technological and molecular advances must be tailored to regional and local needs and resources, with a robust local plan to improve services in a stepwise fashion. This would be more beneficial to children and their families than trying to introduce complex testing with treatment options that the local infrastructure cannot deliver. Although this article has focused on children with CNS tumors, the same principles and issues are present in children with other tumor types and investment in genomic advances as outlined above will benefit all children with cancer.

In conclusion, genomic advances have had a major impact in HICs but much less so in LMICs. To address this imbalance and to continue to improve the outcome of children with CNS tumors in LMICs, stepwise advances in the implementation of molecular diagnostics and the introduction of a widening range of targeted therapies will require continuous major efforts of the worldwide pediatric oncology community including high-level political pressure.

## APPENDIX 1. Examples of How Biology Can Affect Treatment

### Low-Grade Glioma

The main approach to low-grade glioma (LGG) management is safe complete surgical resection if possible. If there is a risk of neurologic morbidity and only biopsy or subtotal resection is possible, then chemotherapy is used. Knowing the molecular markers may allow utilization of targeted therapy as first-line therapy or at progression if they can be accessed through clinical trials or compassionate use programs. It may also assist in discussing future treatment options.

One such example is a *BRAF v600e*–mutated LGG; it is best practice to achieve a gross total resection in an attempt to avoid future risk (although small) of malignant transformation particularly if CDKN2A is deleted and attempt to avoid radiotherapy because of increased risk of transformation.^46^

### Medulloblastoma

Medulloblastoma is the commonest malignant brain tumor of childhood with survival rates in low- and middle-income countries (LMICs) suggesting an urgent need to improve diagnosis and treatment. Timely referral to specialized centers for surgery and MDT discussion can make a difference with correct risk stratification and management and assist with preventing delays in appropriate imaging and radiotherapy planning, which can influence outcomes. Advances in molecular classification allow us to better understand the behavior of medulloblastoma.^16,24,47^ Unfortunately, these findings have not been accompanied by new treatments that can limit the adverse effects of chemotherapy and radiotherapy.

In countries with limited resources, it is possible to seek low-cost, high-impact strategies to achieve the minimum molecular classification, which may allow stratification and consequent reduction in treatment in those with low-risk disease.^48^ Management in high-income country and LMIC settings is shown in Table 5.

### Genetic Factors/Familial Disease That Alter Therapies

Cancer predisposition syndromes (CPSs) in the context of childhood brain tumors play a major role in both optimizing the right therapy and allowing for early detection of cancer and in genetic counseling to other family members. In the past, recognition of CPS was mainly achieved by detailed personal and family history accompanied by physical examination. The availability of tumor genomic testing has enabled the uncovering of patients with CPS as tumor mutations can be traced to the germline. The prevalence of CPS can be as high as 100% in patients with subependymal giant cell astrocytoma in tuberous sclerosis and hemangioblastoma in Von Hippel-Lindau syndrome patients. By contrast, CPSs are exceedingly rare in ependymoma. Importantly, in gliomas, which are the most common childhood brain tumor, CPS such as NF1, MMRD, and Li-Fraumeni can account to a significant amount of tumors and affect their management. In some tumors, the presence of specific mutations results in a mandatory genetic testing. Examples include *TP53* mutations in CPC and SHH medulloblastomas, *SMARCB1* in young children with ATRT, and the mismatch repair genes in gliomas and medulloblastomas.^49^

A diagnosis of CPS in a child should prompt immediate discussion with the family on installment of surveillance and early detection, which improves the outcome higher than any type of therapy when the cancer is already symptomatic. Surveillance protocols have been implemented in Li-Fraumeni syndrome (LFS), constitutional mismatch repair deficiency, rhabdoid predisposition, and several phacomatoses. Modified protocols are built to fit LMICs and can still improve outcomes when focusing on specific tumor types or less expensive tests. An example includes the improved outcome in constitutional mismatch repair deficiency children even when expensive tests such as total body MRI are not used.^49^

The presence of aCPS diagnosis has predictive value. For example, NF1-associated LGG has superior progression-free survival and will rarely cause death. As such, aggressive therapies should not be considered. By contrast, CPC and medulloblastoma in the context of LFS confer extremely poor outcomes.

In some CPS, specific treatment should be avoided: radiation therapy and alkylators can cause unacceptable toxicities in cases of homozygous mutations in syndromes that carry defective homologous recombination repair. Temozolomide and mercaptopurines will lack any effective impact on tumors driven by MMRD. Radiation therapy may cause secondary malignancies in patients with LFS and NF1. Finally, gliomas in patients with NF1 and MMRD respond dramatically to targeted therapy and immunotherapy, respectively.^50^

Although tumor genomic analysis is still expensive and requires expertise, a combination of current available clinical algorithms and apps as well as simple methods such as immunohistochemistry can eliminate most patients, while suggesting further investigations in the relevant high-risk patients. Final genetic diagnosis can include sending tissues to central laboratories in a carefully selected small subset of patients, which exist in several international consortia. These consortia can offer genetic counseling and guidelines for surveillance and tumor management. Simpler cheap and robust tests such as immunostains for *TP53*, the MMR genes, SmarcB1, and others. If a child has positive stain for *TP53* in CPC or SHH medulloblastoma, the chance of LFS is 20%-50% and genetic testing should be initiated. Indeed, in some cases, survival of patients with CPS can be dramatically affected by such an approach.^49^

## References

[1] Louis DN, Perry A, Wesseling P, et al: The 2021 WHO classification of tumors of the central nervous system: A summary. Neuro Oncol 23:1231-1251, 2021 34185076 10.1093/neuonc/noab106 PMC8328013

[2] Lam CG, Howard SC, Bouffet E, Pritchard-Jones K: Science and health for all children with cancer. Science 363:1182-1186, 2019 30872518 10.1126/science.aaw4892

[3] Moreira DC, Rajagopal R, Navarro-Martin Del Campo RM, et al: Bridging the gap in access to care for children with CNS tumors worldwide. JCO Glob Oncol 6:583-584, 2020 32293939 10.1200/GO.20.00047 PMC7193818

[4] Seah T, Zhang C, Halbert J, et al: The magnitude and predictors of therapy abandonment in pediatric central nervous system tumors in low- and middle-income countries: Systematic review and meta-analysis. Pediatr Blood Cancer 66:e27692, 2019 30835958 10.1002/pbc.27692

[5] Allemani C, Matsuda T, Di Carlo V, et al: Global surveillance of trends in cancer survival 2000-14 (CONCORD-3): Analysis of individual records for 37 513 025 patients diagnosed with one of 18 cancers from 322 population-based registries in 71 countries. Lancet 391:1023-1075, 2018 29395269 10.1016/S0140-6736(17)33326-3 PMC5879496

[6] Elhassan MMA, Mohamedani AA, Osman HHM, et al: Patterns, treatments, and outcomes of pediatric central nervous system tumors in Sudan: A single institution experience. Childs Nerv Syst 35:437-444, 2019 30610484 10.1007/s00381-018-04032-9

[7] Crosier S, Hicks D, Schwalbe EC, et al: Advanced molecular pathology for rare tumours: A national feasibility study and model for centralised medulloblastoma diagnostics. Neuropathol Appl Neurobiol 47:736-747, 2021 33826763 10.1111/nan.12716 PMC8600954

[8] WHO Model Lists of Essential Medicines. https://www.who.int/groups/expert-committee-on-selection-and-use-of-essential-medicines/essential-medicines-lists

[9] Ocran Mattila P, Ahmad R, Hasan SS, Babar ZU: Availability, affordability, access, and pricing of anti-cancer medicines in low- and middle-income countries: A systematic review of literature. Front Public Health 9:628744, 2021 33996712 10.3389/fpubh.2021.628744 PMC8120029

[10] WHO: Technical Report: Pricing of Cancer Medicines and its Impacts: A Comprehensive Technical Report for the World Health Assembly Resolution 70.12: Operative Paragraph 2.9 on Pricing Approaches and Their Impacts on Availability and Affordability of Medicines. World Health Organization, Geneva, Switzerland, 2018

[11] Fundytus A, Sengar M, Lombe D, et al: Access to cancer medicines deemed essential by oncologists in 82 countries: An international, cross-sectional survey. Lancet Oncol 22:1367-1377, 2021 34560006 10.1016/S1470-2045(21)00463-0 PMC8476341

[12] Pérez JPM, Muchart J, López VS, et al: Targeted therapy for pediatric low-grade glioma. Childs Nerv Syst 37:2511-2520, 2021 33864514 10.1007/s00381-021-05138-3

[13] Manoharan N, Choi J, Chordas C, et al: Trametinib for the treatment of recurrent/progressive pediatric low-grade glioma. J Neurooncol 149:253-262, 2020 32780261 10.1007/s11060-020-03592-8

[14] Cuomo RE, Seidman RL, Mackey TK: Country and regional variations in purchase prices for essential cancer medications. BMC Cancer 17:566, 2017 28836947 10.1186/s12885-017-3553-5 PMC5571501

[15] Northcott PA, Robinson GW, Kratz CP, et al: Medulloblastoma. Nat Rev Dis Primers 5:11, 2019 30765705 10.1038/s41572-019-0063-6

[16] Northcott PA, Korshunov A, Witt H, et al: Medulloblastoma comprises four distinct molecular variants. J Clin Oncol 29:1408-1414, 2011 20823417 10.1200/JCO.2009.27.4324 PMC4874239

[17] Schwalbe EC, Hicks D, Rafiee G, et al: Minimal methylation classifier (MIMIC): A novel method for derivation and rapid diagnostic detection of disease-associated DNA methylation signatures. Sci Rep 7:13421, 2017 29044166 10.1038/s41598-017-13644-1 PMC5647382

[18] Li M, Lee KF, Lu Y, et al: Frequent amplification of a chr19q13.41 microRNA polycistron in aggressive primitive neuroectodermal brain tumors. Cancer Cell 16:533-546, 2009 19962671 10.1016/j.ccr.2009.10.025 PMC3431561

[19] Ellison DW, Dalton J, Kocak M, et al: Medulloblastoma: Clinicopathological correlates of SHH, WNT, and non-SHH/WNT molecular subgroups. Acta Neuropathol 121:381-396, 2011 21267586 10.1007/s00401-011-0800-8 PMC3519926

[20] Tauziède-Espariat A, Huybrechts S, Indersie E, et al: Diagnostic accuracy of a reduced immunohistochemical panel in medulloblastoma molecular subtyping, correlated to DNA-methylation analysis. Am J Surg Pathol 45:558-566, 2021 33323893 10.1097/PAS.0000000000001640

[21] Kaur K, Kakkar A, Kumar A, et al: Integrating molecular subclassification of medulloblastomas into routine clinical practice: A simplified approach. Brain Pathol 26:334-343, 2016 26222673 10.1111/bpa.12293 PMC8029101

[22] Korshunov A, Sturm D, Ryzhova M, et al: Embryonal tumor with abundant neuropil and true rosettes (ETANTR), ependymoblastoma, and medulloepithelioma share molecular similarity and comprise a single clinicopathological entity. Acta Neuropathol 128:279-289, 2014 24337497 10.1007/s00401-013-1228-0 PMC4102829

[23] Finetti MA, Grabovska Y, Bailey S, Williamson D: Translational genomics of malignant rhabdoid tumours: Current impact and future possibilities. Semin Cancer Biol 61:30-41, 2020 31923457 10.1016/j.semcancer.2019.12.017

[24] Ramaswamy V, Remke M, Bouffet E, et al: Risk stratification of childhood medulloblastoma in the molecular era: The current consensus. Acta Neuropathol 131:821-831, 2016 27040285 10.1007/s00401-016-1569-6 PMC4867119

[25] Gessi M, Giagnacovo M, Modena P, et al: Role of immunohistochemistry in the identification of supratentorial C11ORF95-RELA fused ependymoma in routine neuropathology. Am J Surg Pathol 43:56-63, 2019 29266023 10.1097/PAS.0000000000000979

[26] Panwalkar P, Clark J, Ramaswamy V, et al: Immunohistochemical analysis of H3K27me3 demonstrates global reduction in group-A childhood posterior fossa ependymoma and is a powerful predictor of outcome. Acta Neuropathol 134:705-714, 2017 28733933 10.1007/s00401-017-1752-4 PMC5647236

[27] Ghasemi DR, Sill M, Okonechnikov K, et al: MYCN amplification drives an aggressive form of spinal ependymoma. Acta Neuropathol 138:1075-1089, 2019 31414211 10.1007/s00401-019-02056-2 PMC6851394

[28] Parkes J, Hendricks M, Ssenyonga P, et al: SIOP PODC adapted treatment recommendations for standard-risk medulloblastoma in low and middle income settings. Pediatr Blood Cancer 62:553-564, 2015 25418957 10.1002/pbc.25313

[29] Hessissen L, Parkes J, Amayiri N, et al: SIOP PODC Adapted treatment guidelines for low grade gliomas in low and middle income settings. Pediatr Blood Cancer 64:e26737, 2017 (suppl 5) 10.1002/pbc.26737 29297618

[30] Amayiri N, Spitaels A, Zaghloul M, et al: SIOP PODC-adapted treatment guidelines for craniopharyngioma in low- and middle-income settings. Pediatr Blood Cancer:e28493, 2020 32790146 10.1002/pbc.28493

[31] Azad TD, Shrestha RK, Vaca S, et al: Pediatric central nervous system tumors in Nepal: Retrospective analysis and literature review of low- and middle-income countries. World Neurosurg 84:1832-1837, 2015 26283488 10.1016/j.wneu.2015.07.074

[32] Lopez-Aguilar E, Sepulveda-Vildosola AC, Rivera-Marquez H, et al: Survival of patients with medulloblastoma treated with carboplatin and etoposide before and after radiotherapy. Arch Med Res 29:313-317, 1998 9887549

[33] Salama MM, Ghorab EM, Al-Abyad AG, Al-Bahy KM: Concomitant weekly vincristine and radiation followed by adjuvant vincristine and carboplatin in the treatment of high risk medulloblastoma: Ain Shams University Hospital and Sohag Cancer Center study. J Egypt Natl Canc Inst 18:167-174, 2006 17496943

[34] Qaddoumi I, Musharbash A, Elayyan M, et al: Closing the survival gap: Implementation of medulloblastoma protocols in a low-income country through a twinning program. Int J Cancer 122:1203-1206, 2008 17985345 10.1002/ijc.23160

[35] Gupta T, Sinha S, Chinnaswamy G, et al: Safety and efficacy of concurrent carboplatin during full-dose craniospinal irradiation for high-risk/metastatic medulloblastoma in a resource-limited setting. Pediatr Blood Cancer 68:e28925, 2021 33533557 10.1002/pbc.28925

[36] Amayiri N, Swaidan M, Ibrahimi A, et al: Molecular subgroup is the strongest predictor of medulloblastoma outcome in a resource-limited country. JCO Glob Oncol 7:1442-1453, 2021 34609903 10.1200/GO.21.00127 PMC8492378

[37] Kaur K, Jha P, Pathak P, et al: Approach to molecular subgrouping of medulloblastomas: Comparison of NanoString nCounter assay versus combination of immunohistochemistry and fluorescence in-situ hybridization in resource constrained centres. J Neurooncol 143:393-403, 2019 31104222 10.1007/s11060-019-03187-y

[38] Rajagopal R, Abd-Ghafar S, Ganesan D, et al: Challenges of treating childhood medulloblastoma in a country with limited resources: 20 years of experience at a single tertiary center in Malaysia. JCO Glob Oncol 3:143-156, 2017 10.1200/JGO.2015.002659 PMC5493270 28717752

[39] Amayiri N, Swaidan M, Al-Hussaini M, et al: Sustained response to targeted therapy in a patient with disseminated anaplastic pleomorphic xanthoastrocytoma. J Pediatr Hematol Oncol 40:478-482, 2018 29200156 10.1097/MPH.0000000000001032

[40] Mustansir F, Mushtaq N, Darbar A: Dabrafenib in BRAFV600E mutant pilocytic astrocytoma in a pediatric patient. Childs Nerv Syst 36:203-207, 2020 31418082 10.1007/s00381-019-04346-2

[41] Hopkins J, Burns E, Eden T: International twinning partnerships: An effective method of improving diagnosis, treatment and care for children with cancer in low-middle income countries. J Cancer Policy 1:e8-e19, 2013

[42] Mushtaq N, Mustansir F, Minhas K, et al: Building the ecosystem for pediatric neuro-oncology care in Pakistan: Results of a 7-year long twinning program between Canada and Pakistan. Pediatr Blood Cancer 69:e29726, 2022 35484912 10.1002/pbc.29726

[43] Global initiative for childhood cancer: https://www.who.int/publications/m/item/global-initiative-for-childhood-cancer

[44] Major A, Palese M, Ermis E, et al: Mapping pediatric oncology clinical trial collaborative groups on the global stage. JCO Glob Oncol 8:e2100266, 2022 35157510 10.1200/GO.21.00266 PMC8853619

[45] Kanwar VS, Schwartz KR, Salifu N, et al: The role of twinning in sustainable care for children with cancer: A TIPPing point? SIOP PODC Working Group on Twinning, Collaboration, and Support. Pediatr Blood Cancer 67:e28667, 2020 32827347 10.1002/pbc.28667

[46] Lassaletta A, Zapotocky M, Mistry M, et al: Therapeutic and prognostic implications of BRAF V600E in pediatric low-grade gliomas. J Clin Oncol 35:2934-2941, 2017 28727518 10.1200/JCO.2016.71.8726 PMC5791837

[47] Taylor MD, Northcott PA, Korshunov A, et al: Molecular subgroups of medulloblastoma: The current consensus. Acta Neuropathol 123:465-472, 2012 22134537 10.1007/s00401-011-0922-z PMC3306779

[48] Epelman S, Sakamoto LH, Real JM, et al: Mbrs-66. Cost-effective method to incorporate molecular classification of medulloblastoma into a Latin-American clinical trial. Neuro-Oncology 23:iii409-iii410, 2020

[49] Tabori U, Hansford JR, Achatz MI, et al: Clinical management and tumor surveillance recommendations of inherited mismatch repair deficiency in childhood. Clin Cancer Res 23:e32-e37, 2017 28572265 10.1158/1078-0432.CCR-17-0574

[50] Bouffet E, Larouche V, Campbell BB, et al: Immune checkpoint inhibition for hypermutant glioblastoma multiforme resulting from germline biallelic mismatch repair deficiency. J Clin Oncol 34:2206-2211, 2016 27001570 10.1200/JCO.2016.66.6552

