# Erratum

**DOI:** 10.1200/GO.22.00400

**Published:** 2023-01-06

The article by Bailey et al entitled “How Can Genomic Innovations in Pediatric Brain Tumors Transform Outcomes in Low- and Middle-Income Countries?” (*JCO Glob Oncol* 10.1200/GO.22.00156) was published online October 17, 2022, with an error.

The eighth coauthor's name was misspelled in the author list and author contributions. Rosaldi Dias-Coronado should have read as **Rosdali Diaz-Coronado**.

This has been corrected as of January 6, 2023. The authors apologize for the error.

